# Hybrid Value-Aware Transformer Identifies Novel and Suspected Drugs for Alzheimer’s Disease

**DOI:** 10.1093/geroni/igaf122.3958

**Published:** 2025-12-31

**Authors:** Qing Zeng, Yijun Shao, Ying Yin, Edward Zamrini, Ali Ahmed, Cheng Yan

**Affiliations:** The George Washington University, Washington, District of Columbia, United States; George Washington University, Washington, DC, Washington, District of Columbia, United States; George Washington University, George Washington University, District of Columbia, United States; Irvine Clinical Research, Irvine, California, United States; Washington DC VA Medical Center, Washington, District of Columbia, United States; George Washington University, Washington, District of Columbia, United States

## Abstract

Drug discovery for Alzheimer’s disease and related dementias (ADRD) faces many challenges including the complex and multifactorial nature of ADRD. Our research team developed the Hybrid Value-Aware Transformer (HVAT), a novel deep learning architecture designed to model multimodal health data. HVAT extends Transformer models, a powerful AI technology originally developed for natural language processing and generation, with “clinical tokens” that represent both longitudinal and non-longitudinal data, while also incorporating numerical values such as laboratory results and cumulative drug doses. We used this hybrid, value-aware model to identify dose-dependent associations between drug exposures and ADRD risk. Specifically, we trained an HVAT model on a case-control cohort of approximately one million U.S. Veterans aged 65 years and older, with up to 10 years of medical history. HVAT achieved fair predictive performance (area under the curve [AUC] = 0.775) in distinguishing ADRD cases from controls. We then derived population-level drug impact scores, a novel explainable AI measure similar to odds ratio, to quantify the association between normalized cumulative dose and ADRD risk. Our analyses yielded both suspected candidates, such as varenicline, which has supportive preclinical and early clinical evidence in ADRD, and novel candidates, such as metolazone, a thiazide-like diuretic not previously linked to dementia. These findings demonstrate that HVAT can recover known signals while also uncovering new drug repurposing opportunities. By combining deep learning with explainable AI analysis, this project represents a generalizable AI framework for accelerating drug discovery in aging research.

